# Successful Robot‐Assisted Resection of a Retroperitoneal Cystic Tumor With Fluorescent Ureteral Stent and Firefly Imaging

**DOI:** 10.1002/iju5.70125

**Published:** 2025-12-12

**Authors:** Yoichiro Tohi, Kana Kohashiguchi, Kenichi Tanaka, Mari Jinno, Takuma Kato, Rikiya Taoka, Nobufumi Ueda, Reiji Haba, Yoshihiro Nishiyama, Mikio Sugimoto

**Affiliations:** ^1^ Department of Urology, Faculty of Medicine Kagawa University Takamatsu Kagawa Japan; ^2^ Department of Radiology, Faculty of Medicine Kagawa University Takamatsu Kagawa Japan; ^3^ Department of Diagnostic Pathology, Faculty of Medicine Kagawa University Takamatsu Kagawa Japan

**Keywords:** firefly imaging, fluorescent ureteral catheter, retroperitoneal tumor, robot‐assisted resection, ureteral injury

## Abstract

**Introduction:**

We present a technique that combines a fluorescent ureteral catheter with Firefly near‐infrared fluorescence imaging to identify the ureter and avoid injury during robot‐assisted resection of a retroperitoneal cystic tumor.

**Case Presentation:**

Computed tomography revealed a 6‐cm retroperitoneal cystic mass anterior to the abdominal aorta near the lower pole of the left kidney that adhered to the left ureter in a 53‐year‐old man. A fluorescent ureteral catheter was placed preoperatively, and robot‐assisted transperitoneal resection was performed. Firefly imaging enabled clear visualization of the ureter, allowing safe dissection and tumor identification. The tumor was excised without cyst rupture. The console time was 4 h 13 min, with minimal blood loss. Pathology confirmed schwannoma.

**Conclusion:**

We demonstrated the feasibility and clinical benefits of integrating a fluorescent ureteral catheter with Firefly fluorescence imaging for intraoperative ureteral identification during robot‐assisted surgery. This fluorescence‐guided approach improves dissection accuracy and helps prevent ureteral injury.

## Keynote Message

The combination of a fluorescent ureteral catheter and Firefly near‐infrared imaging enables real‐time visualization of the ureter during robot‐assisted surgery. This approach allows safe dissection of a cystic retroperitoneal tumor while avoiding ureteral injury.

## 1 Introduction

Retroperitoneal tumors attached to the ureter and major vessels pose a risk of iatrogenic injury during surgery. Intraoperative identification of the ureter is critical; however, traditional methods (preoperative imaging, palpation, or visible catheters) can be unreliable. In recent years, there has been a shift toward minimally invasive surgery, such as robot‐assisted surgery, and the importance of visual information during surgery has increased.

Fluorescence‐guided techniques have enhanced the identification of key anatomical landmarks, such as ureteral visualization [1]. For example, fluorescent ureteral catheters have been used in laparoscopic surgery to highlight the ureter in real time [2, 3, 4, 5, 6]. These catheters emit light in the near‐infrared (NIR) spectrum and can be visualized with laparoscopic fluorescence systems, greatly improving ureteral visibility.

In robotic surgery, the da Vinci Firefly system (Intuitive Surgical Inc., Sunnyvale, CA, USA) provides integrated NIR fluorescence imaging from the surgeon's perspective. A previous report showed that NIR enhances real‐time ureteral visualization and reduces the risk of iatrogenic injury during complex pelvic surgery [7].

Herein, we report a case in which the combined use of a fluorescent ureteral catheter and Firefly NIR fluorescence imaging enabled precise intraoperative identification of the ureter and facilitated its preservation during robot‐assisted resection of a retroperitoneal cystic tumor.

## 2 Case Presentation

**FIGURE 1:**
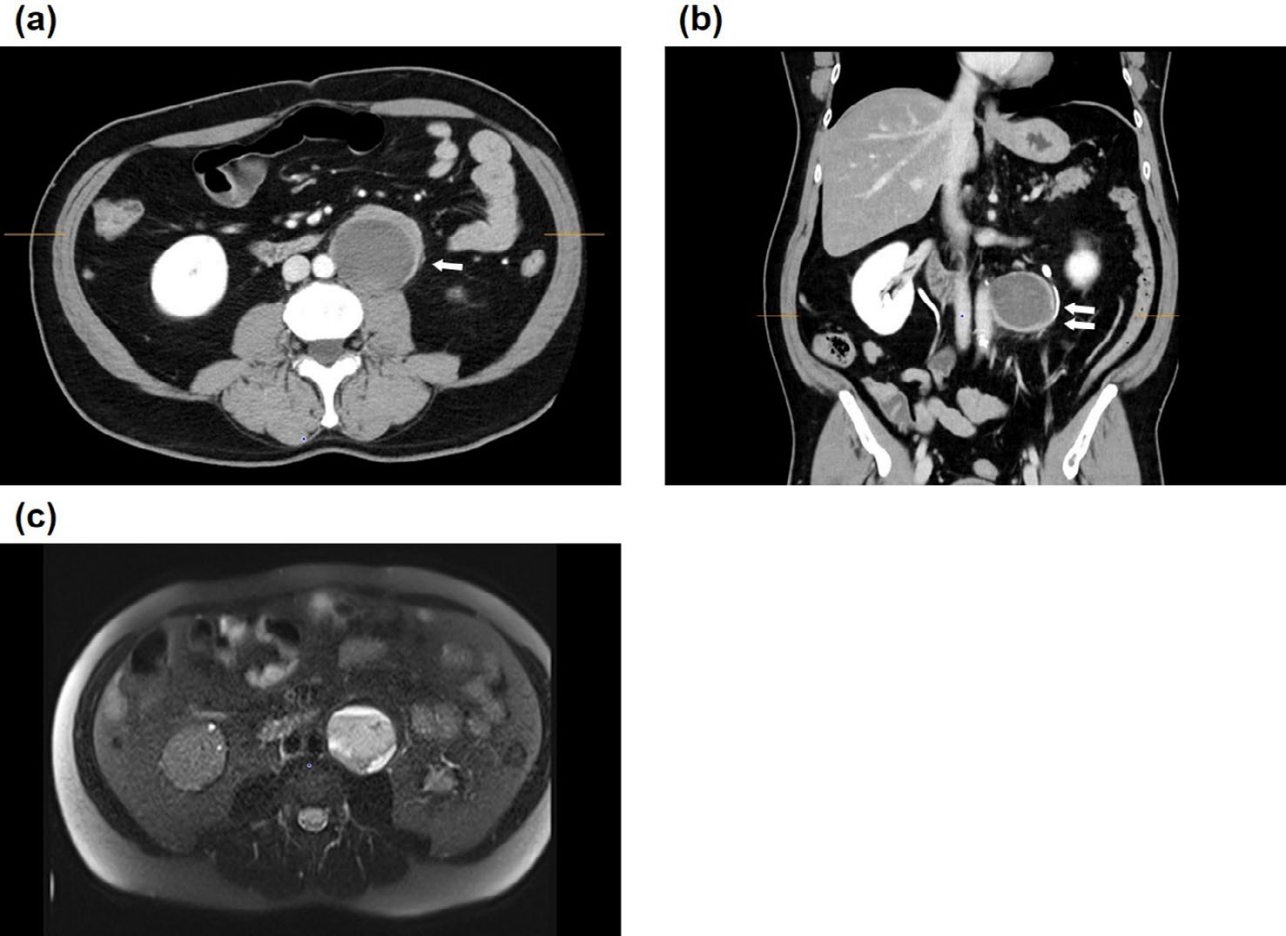
Abdominal CT and MRI findings. (a) Contrast‐enhanced CT scan showing a cystic mass in the left retroperitoneum, adherent to the abdominal aorta. The tumor measured 6.5 cm in size. A peripheral solid component of the cystic mass demonstrated progressive contrast enhancement. The left ureter (arrows) was adherent to the tumor (axial view). (b) On the excretory phase of contrast‐enhanced CT, the left ureter (arrows) was observed to be adherent to the tumor (coronal view). (c) On T2‐weighted MRI, the lesion exhibited high signal intensity, with a cystic component. CT, computed tomography; MRI, magnetic resonance imaging.

A 53‐year‐old man was evaluated for incidental left flank fullness on imaging. Computed tomography (CT) revealed a cystic mass in the left retroperitoneum that was adhered to the left ureter and abdominal aorta (Figure 1a,b). The tumor measured 6.5 × 6.0 × 5.1 cm and showed contrast enhancement (Figure 1a,b). The tumor did not appear invasive, but its location raised concerns regarding potential nerve sheath tumors or other retroperitoneal pathologies. Endocrinological evaluation revealed no evidence of hormone secretion, and iodine‐123 metaiodobenzylguanidine scintigraphy revealed no abnormal tracer accumulation. The patient had no history of surgery. The patient's height, weight, and body mass index were 162.2 cm, 69.4 kg, and 26.38 kg/m^2^, respectively. To aid intraoperative ureteral identification, a 6‐Fr ureteral catheter containing a fluorescent dye that emits fluorescence (Near Infrared Ray Catheter [NIRC]; Cardinal Health K.K., Fukuoka, Japan) was retrogradely placed in the left ureter, under cystoscopic guidance, immediately before surgery.

**FIGURE 2:**
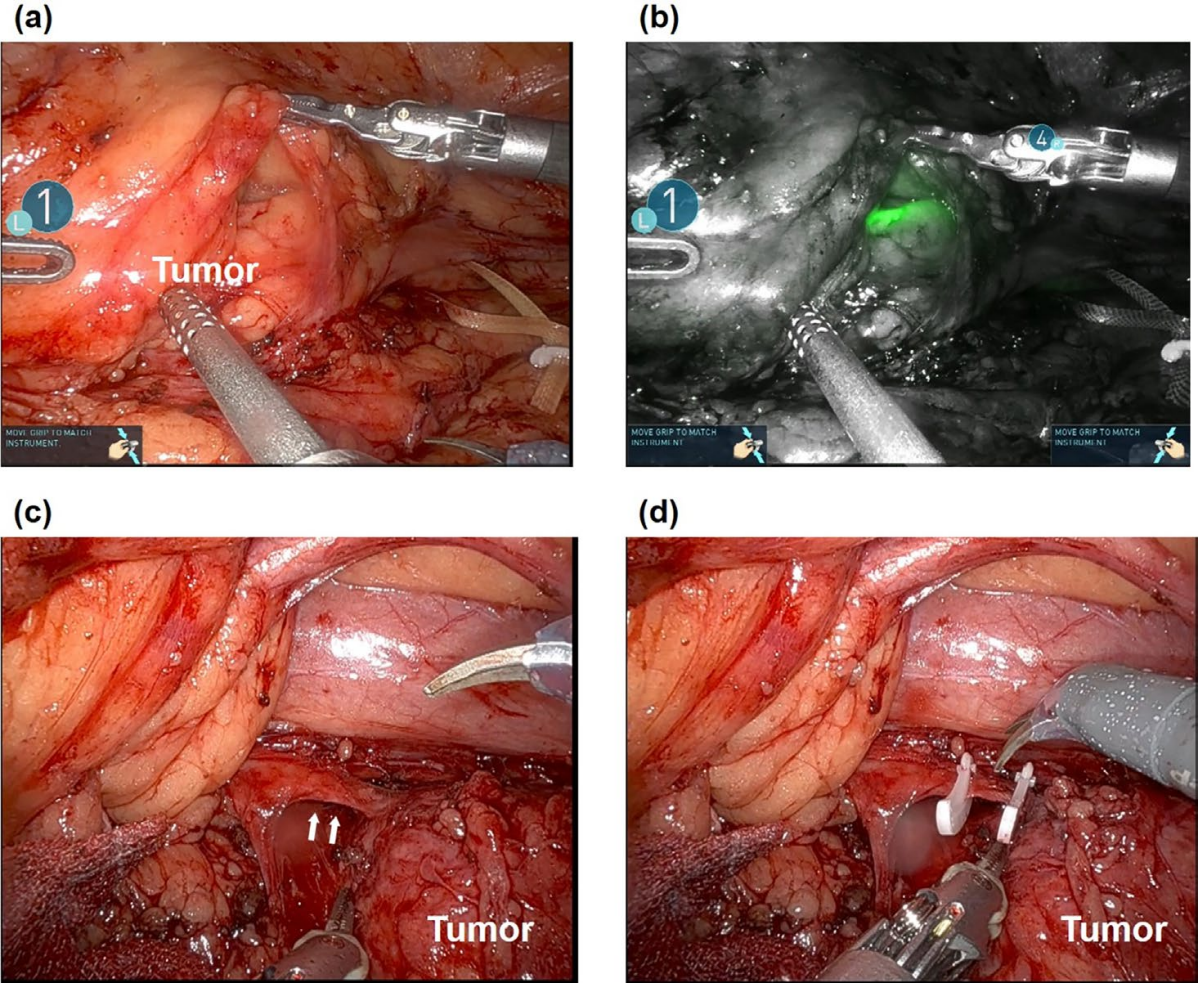
Intraoperative findings. The left ureter was clearly identified using Firefly near‐infrared fluorescence imaging and was found to be adherent to the tumor. (a) White light imaging. (b) Near‐infrared fluorescence imaging. (c) The cranial aspect of the tumor is continuous with the sympathetic trunk (arrows). (d) The sympathetic trunk is separated from the tumor using hemoclips.

**FIGURE 3:**
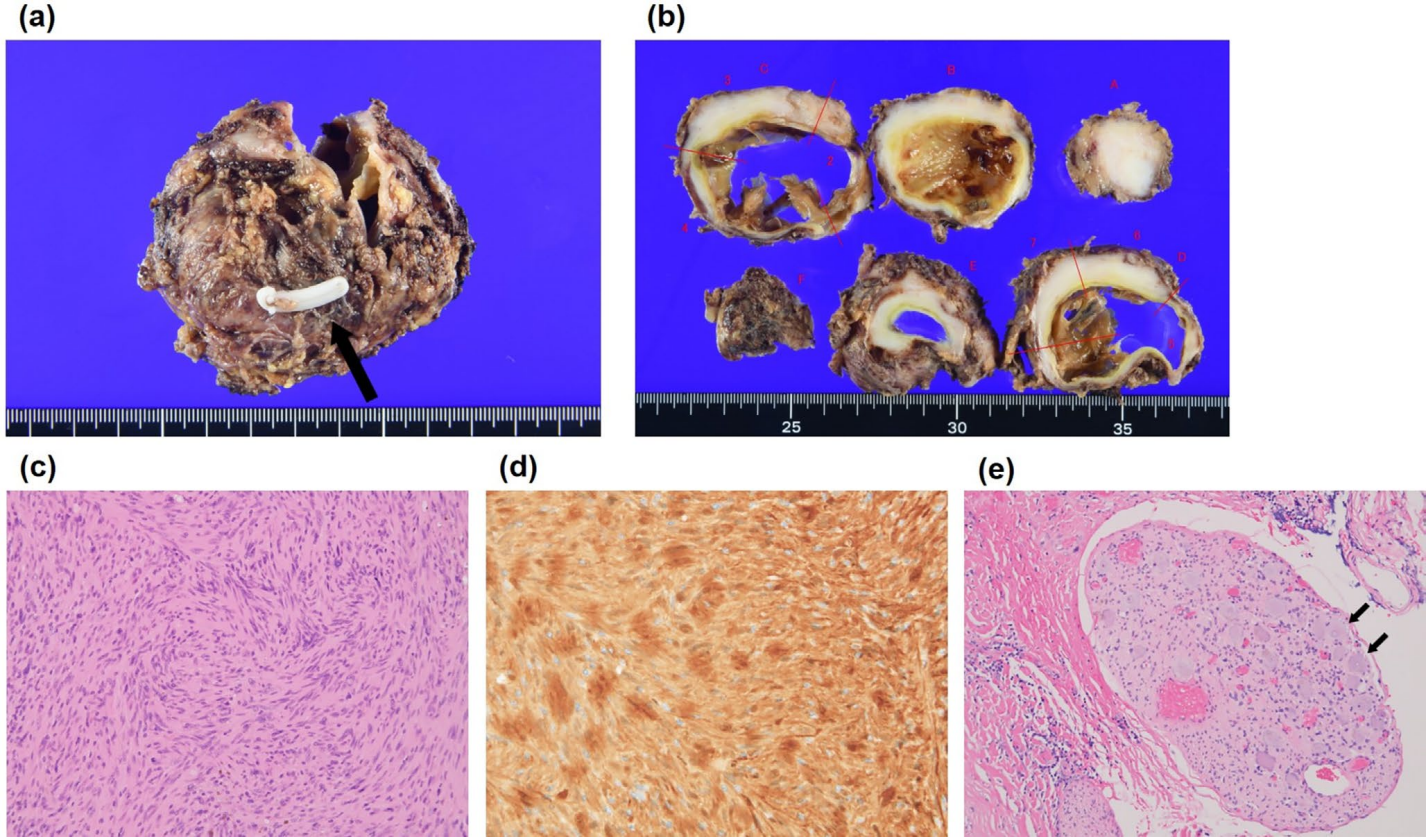
Gross and microscopic findings. (a) The tumor measured 6.5 cm, and an intraoperative clip was observed attached to the sympathetic trunk (arrow). (b) The cystic component with internal septations was identified within the lesion. (c) Hematoxylin and eosin staining revealed proliferation of tumor cells with elongated cytoplasm (original magnification ×200). (d) Immunostaining for S‐100 protein was positive (original magnification ×400). (e) Ganglion cells were also observed at the edge of the tumor (original magnification ×200) (arrows).

The procedure was performed via a transperitoneal approach, with port placement similar to that used for robot‐assisted transperitoneal nephrectomy. The mesocolon was densely adherent to the tumor; therefore, the descending colon was mobilized caudally to access the lesion. After exposing the psoas muscle, the ureter was identified and dissected cranially; however, dissection on the caudal side of the tumor was challenging because of dense tissue adhesion. Intraoperative ultrasonography was performed to confirm the exact location of the tumor, after which circumferential dissection around the tumor was performed. Firefly NIR fluorescence imaging was subsequently used to identify the ureter that was adhered to the tumor, which was preserved, and circumferential dissection was performed around the tumor located anterior to the abdominal aorta. The cranial aspect of the tumor was continuous with the sympathetic trunk, which was clipped and separated using hemoclips (Figure 2c,d). The tumor was excised en bloc, along with the adherent mesocolon, without rupturing the cyst wall. Dissection was performed around the inferior mesenteric artery, and colonic perfusion was assessed intraoperatively using Firefly fluorescence imaging at the end of the procedure. The tumor was placed in an E･Z PURSE (Hakko Medical, Nagano, Japan) and externally extracted. The console time was 4 h 13 min, with minimal blood loss. Pathological examination confirmed schwannoma (Figure 3a,e). A postoperative grade 2 chyle leak was observed, which improved with fasting and octreotide therapy. The patient was discharged on postoperative day 13 and has remained recurrence‐free for 6 months postoperatively.

## 3 Discussion

In this case, we demonstrated the successful utilization of a fluorescent ureteral catheter with the da Vinci Firefly NIR imaging system to safely resect a retroperitoneal cystic tumor. Preoperative imaging indicated that the 6‐cm cystic mass was adherent to the left ureter; therefore, a fluorescent ureteral catheter was placed to highlight the ureter intraoperatively. This allowed continuous real‐time visualization of the ureter throughout the robotic dissection, enabling a smooth approach to the tumor and precise excision without ureteral injury. The ureter was clearly identified and preserved despite its dense attachment, and the schwannoma was removed. This case illustrates that fluorescent ureteral stenting combined with integrated NIR imaging can be feasibly performed in complex retroperitoneal surgeries.

Ureter identification is critical in pelvic and retroperitoneal operations because inadvertent ureteral injuries, although infrequent (with an incidence ranging from approximately 0.5%–1%), can cause significant morbidity if unrecognized [8]. The risk of iatrogenic ureteral injury is heightened during minimally invasive surgery owing to reduced tactile feedback and extensive use of energy devices [8]. Fluorescence‐guided ureter visualization may offer a strategy to avoid injury [9]. The Firefly NIR fluorescence imaging system integrated into the da Vinci robotic platform enables real‐time visualization of indocyanine green fluorescence in the operative field. No additional equipment is required for its use. This system is a standard feature of the da Vinci. In our case, the glowing ureteral catheter served as a road map for dissection. This experience aligns with prior reports [3, 4, 5, 6]. This technique effectively compensates for the visual limitations of conventional methods, such as landmark placement of the ureteral stent. Considering its safety and utility, we believe that the proactive use of a fluorescent ureteral catheter should be considered in surgeries with a significant risk of ureteral injury, such as reoperations, to improve intraoperative guidance. In addition to preventing direct mechanical injury, fluorescent ureteral stenting may reduce the risk of ischemic ureteral injury by facilitating more precise dissection with minimal traction or thermal spread near the ureter. Because it provides real‐time visual feedback, surgeons can avoid excessive manipulation or inadvertent coagulation, which may compromise the ureteral blood supply. This may be particularly beneficial in cases where the ureter is densely adherent to tumor tissue, as observed in the present case.

Schwannomas are benign tumors of Schwann cell origin that rarely occur in the retroperitoneum, accounting for only 0.5%–5% of all schwannoma cases [10]. They are usually encapsulated and arise from the peripheral nerves. In our case, the tumor was connected to the nerve bundle during surgery, likely arising from the sympathetic trunk. Fortunately, no postoperative neuropathy or pain was observed in our case; however, permanent nerve damage has been reported to occur in a certain percentage of cases [11].

In conclusion, we reported the feasibility and clinical benefits of combining a fluorescent ureteral catheter with Firefly NIR imaging for robot‐assisted resection of a retroperitoneal cystic tumor attached to the ureter. This fluorescence‐guided approach provides real‐time ureteral visualization, which enhances dissection accuracy and helps avoid ureteral injury.

## Ethics Statement

This study was approved by the Institutional Review Board of Kagawa University (approval No. 2025‐132). The surgical procedure was reviewed and approved by the hospital's internal committee for advanced surgical techniques.

## Consent

Informed consent was obtained from the patient for the publication of this case report and accompanying images.

## Conflicts of Interest

Mikio Sugimoto is an editorial board member of the *International Journal of Urology* and was thus excluded from all editorial decision‐making related to the acceptance of this article for publication to minimize bias. The rest of the authors declare no conflict of interest.

## Data Availability

The data that support the findings of this study are available from the corresponding author upon reasonable request.
